# Highly Sensitive Colorimetric Assay for Determining Fe^3+^ Based on Gold Nanoparticles Conjugated with Glycol Chitosan

**DOI:** 10.1155/2017/3648564

**Published:** 2017-05-23

**Authors:** Kyungmin Kim, Yun-Sik Nam, Yeonhee Lee, Kang-Bong Lee

**Affiliations:** ^1^Green City Technology Institute, Korea Institute of Science and Technology, Hwarang-ro 14gil 5, Seongbuk-gu, Seoul 02792, Republic of Korea; ^2^Department of Chemistry, Korea University, Anam-ro 145, Seongbuk-gu, Seoul 02841, Republic of Korea; ^3^Advanced Analysis Center, Korea Institute of Science and Technology, Hwarang-ro 14gil 5, Seongbuk-gu, Seoul 02792, Republic of Korea

## Abstract

A highly sensitive and simple colorimetric assay for the detection of Fe^3+^ ions was developed using gold nanoparticles (AuNPs) conjugated with glycol chitosan (GC). The Fe^3+^ ion coordinates with the oxygen atoms of GC in a hexadentate manner (O-Fe^3+^-O), decreasing the interparticle distance and inducing aggregation. Time-of-flight secondary ion mass spectrometry showed that the bound Fe^3+^ was coordinated to the oxygen atoms of the ethylene glycol in GC, which resulted in a significant color change from light red to dark midnight blue due to aggregation. Using this GC-AuNP probe, the quantitative determination of Fe^3+^ in biological, environmental, and pharmaceutical samples could be achieved by the naked eye and spectrophotometric methods. Sensitive response and pronounced color change of the GC-AuNPs in the presence of Fe^3+^ were optimized at pH 6, 70°C, and 300 mM NaCl concentration. The absorption intensity ratio (*A*_700_/*A*_510_) linearly correlated to the Fe^3+^ concentration in the linear range of 0–180 *μ*M. The limits of detection were 11.3, 29.2, and 46.0 nM for tap water, pond water, and iron supplement tablets, respectively. Owing to its facile and sensitive nature, this assay method for Fe^3+^ ions can be applied to the analysis of drinking water and pharmaceutical samples.

## 1. Introduction

Fe^3+^ is an essential trace metal ion that plays a vital role in living organisms. It is essential to maintain and balance the iron level in our body because both its deficiency and its excess can induce a variety of diseases [1–4]. In this context, a convenient assay method is required, necessitating the design of simple, highly sensitive, and selective sensor for trace level detection of Fe^3+^ in biological and environmental samples.

Numerous analytical methods including inductively coupled plasma atomic emission spectrometry, atomic absorption spectrometry (AAS), and inductively coupled plasma mass spectrometry have recently been utilized to detect ferric ions [5, 6]. However, these methods usually require sophisticated instrumentation, tedious sample pretreatment steps, and well-trained operators. Therefore, development of a facile analytical method for the detection of Fe^3+^ ions is still highly desirable. Recently, numerous fluorescence sensors and chemosensors have been reported for the detection of metal ions; these sensors have several advantages, such as ease of use, high sensitivity, low-cost, and enabling of on-site monitoring [7–16]. Colorimetric nanoparticle assays have also been widely used to monitor metal ions because of their cost-efficiency and applicability for on-site monitoring, as opposed to other methods [17, 18]. Advances in nanoscience and nanotechnology have led to the development of new nanoparticle assays for Fe^3+^ ions detection such as Cu or Au nanoclusters [19, 20], etching [21, 22], graphene quantum dot [23], and carbon dot [24] assay methods. Moreover, nanoparticle-based assay method using ligands that coordinate to Fe^3+^ ions have been recently reported, in which nanoparticles are functionalized with receptors with specific binding affinity for Fe^3+^ ions. Among these, silver nanoparticles (AgNPs) conjugated with a pyridyl-appended calix[4]arene and gold nanoparticles (AuNPs) functionalized with pyrophosphate, histidine, or p-amino salicylic acid dithiocarbamate have recently been developed as Fe^3+^ color sensors [25–28].

**Scheme 1:**
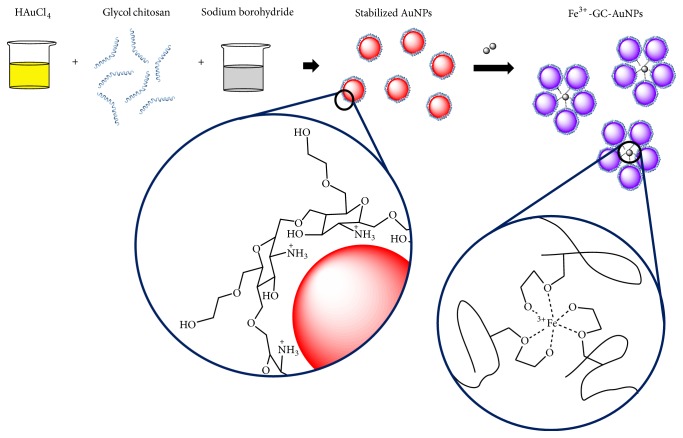
Schematic illustration of a AuNP capped with GC, aggregation of GC-AuNPs reacting with Fe^3+^ ions, accompanied by a color change, and the predicted coordination bond between Fe^3+^ ions and GC-AuNP. AuNPs, gold nanoparticles; GC-AuNPs, gold nanoparticles conjugated with GC.

Glycol chitosan (GC) is a water soluble chitosan derivative, and AuNPs conjugated with GC (GC-AuNPs) have been used in biomedical applications [29, 30]. GC can easily bind to AuNPs through the -NH groups as -SH groups were usually conjugated to AuNPs (Scheme 1). The physicochemical properties of GC could be exploited to develop colorimetric probes for specific metal ion monitoring in aqueous solution, based on the complexing ability of its oxygen atoms with metal ions. The binding sites of GC-AuNPs for Fe^3+^ ions were characterized by time-of-flight secondary ion mass spectrometry (TOF-SIMS) and X-ray photoelectron spectroscopy (XPS).

We show here that GC-AuNPs aggregate upon reaction with Fe^3+^ ions, leading to a color change only observed for Fe^3+^ ions even in the presence of other metal ions. The potential interference effects from other anions were also evaluated. In addition, the optimum conditions, concentration linearity of the reaction, and limit of detection for the GC-AuNP Fe^3+^ sensor were identified. This optimized GC-AuNPs could be exploited in simple and convenient real-time assays for the detection of Fe^3+^ ions.

## 2. Materials and Methods

### 2.1. Materials

Gold(III) chloride trihydrate (HAuCl_4_·3H_2_O), GC, sodium borohydride (NaBH_4_), sodium thiocyanate (NaSCN), sodium cyanide (NaCN), ascorbic acid, sodium hexametaphosphate ((NaPO_3_)_6_), iron(II) sulfate heptahydrate (FeSO_4_·7H_2_O), lithium iron(II) phosphate (LiFePO_4_), and iron disulfide (FeS_2_) were sourced from Sigma-Aldrich (St. Louis, MO, USA). Salts of Fe^3+^, Fe^2+^, Ba^2+^, Mn^2+^, Ga^3+^, Ti^4+^, Al^3+^, Mg^2+^, K^+^, Ag^+^, Ge^4+^, Cr^3+^, Cu^2+^, Li^+^, As^3+^, Co^2+^, Sn^2+^, Na^+^, Pb^2+^, Hg^2+^, Ni^2+^, and Zn^2+^ were purchased from Accu Standard (New Haven, CT, USA). NaCl, HCl, and NaOH were purchased from Samchun Chemical (Gyeong gi-Do, Korea). Distilled water was obtained using a Milli-Q water purification system (Millipore, Bedford, MA, USA). Iron supplement tablets were sourced from Green Cross (Gyeong gi-Do, Korea).

### 2.2. Apparatus

Absorption spectra were recorded on a Sinco S-3100 UV-Vis spectrometer in the range of 300–800 nm using 4-mm path length quartz cuvettes. The pH of the solutions was measured with an HI 2210 pH meter. The concentration of Fe^3+^ ions in aqueous solutions was measured by Varian AAS. A 400 MHz Bruker NMR spectrometer was used to record NMR spectra. Mass spectra were measured using ION-TOF TOF-SIMS. Malvern Zetasizer was used to determine the particle size distribution. CM30 transmission electron microscopy (TEM) and XE-100 atomic force microscopy (AFM) images were obtained from samples prepared by depositing a dispersion of AuNPs and evaporating the solvent.

### 2.3. Preparation of GC-AuNPs

GC-AuNPs were synthesized following literature procedures [29, 30]: 1 mL of HAuCl_4_·3H_2_O (0.025 M) was added to 23 mL of ultrapure water in a round bottom flask with continuous stirring. Then, 7.8 mL of GC (5.64 mM) was added to the stirred solution followed by the slow addition of NaBH_4_ (0.5%), and the solution turned to wine-red. The mixture was stirred overnight at 4°C.

### 2.4. Colorimetric Detection of Fe^3+^ Ions in Real Samples Using Nanoparticles

To evaluate the utility of the method, Fe^3+^ ions in real water samples were determined. Samples were collected from tap and pond water in our research institute (Korea Institute of Science and Technology (KIST)). The results of our method were confirmed by AAS measurements. A syringe filter with pore size of 0.20 *μ*m was used to remove the suspended particles in all water samples. Sample aliquots (9 mL) were mixed with a 100 *μ*gmL^−1^ Fe^3+^ solution (1 mL) to produce a 10 *μ*g mL^−1^ Fe^3+^ concentrated solution. About 0–10 *μ*L of real water samples containing 0–1.0 *μ*gmL^−1^ Fe^3+^ was analyzed using the GC-AuNP solution. The Fe^3+^ content of iron supplement tablets obtained from a local drugstore was quantified. All tablet samples were dissolved in water and centrifuged for 30 min to remove suspended particles. Iron supplements containing 0–20 mg of Fe^3+^ according to manufacturer specifications were examined using 1 mL of the GC-AuNP solution. The analytical results obtained for real samples using GC-AuNP sensing were subsequently confirmed by UV-Vis spectrophotometry.

## 3. Results and Discussion

### 3.1. Characterization of GC-AuNPs and Their Complexes with Fe^3+^

HAuCl_4_·3H_2_O was reduced in the presence of GC using NaBH_4_ as a reducing agent to form GC-AuNPs. The GC-AuNPs obtained under these conditions are much smaller (~10 nm) than those obtained by the conventional citrate-based reduction method (~33 nm). The mean AuNP size is dependent on the amount of reducing agent used and on other conditions [31–35].

**Figure 1:**
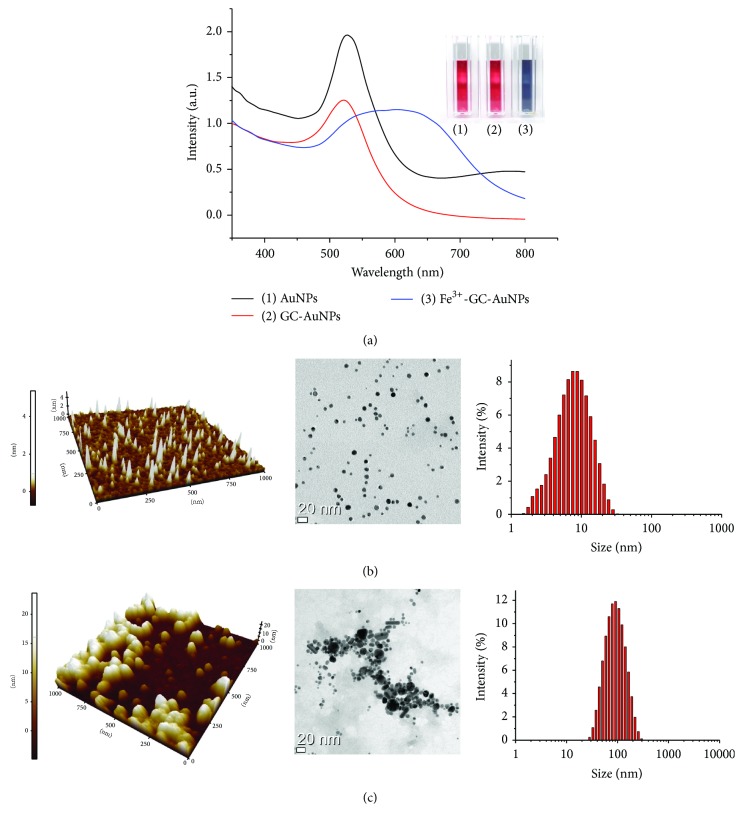
(a) UV-Vis absorption spectra of (1) AuNPs, (2) GC-AuNPs, and (3) GC-AuNPs conjugated with Fe^3+^. (b) 3D-AFM image (left), TEM image (middle), and the corresponding particle size distribution histogram of GC-AuNPs (right). (c) 3D-AFM image (left), TEM image (middle), and particle size distribution histogram of GC-AuNPs conjugated with Fe^3+^ (right). AuNPs, gold nanoparticles; GC-AuNPs, gold nanoparticles conjugated with GC.

The localized surface plasmon resonance (LSPR) maxima and sensing performance of AuNPs depend on the AuNP size. A strong LSPR peak appears at ca. 510 nm in the UV-Vis spectrum of label-free AuNPs, which is associated with the red color of their solutions. Notably, solutions of GC-AuNPs exhibit a similar red color. Thus, it was envisioned that this intense red color could be used to develop a facile assay method for the determination of metal ions. Upon addition of 180 *µ*M Fe^3+^, the color of GC-AuNP solutions was found to rapidly change from red to dark midnight blue (Figure 1(a)), and the intensity of the UV-Vis absorption band at 510 nm decreased with concomitant formation of a new peak at 700 nm. This could be explained by the binding of Fe^3+^ to GC-capped AuNPs, which reduces the internanoparticle distance resulting in a close interaction among GC-AuNPs. The aggregation of GC-AuNPs in the presence of Fe^3+^ ions leads to the delocalization of the surface conduction electrons of AuNPs over neighboring particles, which results in the shift of the absorption band towards lower energies. This phenomenon allows the easy differentiation of Fe^3+^ from other metal ions. AFM and TEM images as well as size distribution measurements of GC-AuNPs and Fe^3+^-GC-AuNPs (Figures 1(b) and 1(c)) revealed that the size of GC-AuNPs and Fe^3+^-GC-AuNPs was ~10 and 102 nm, respectively (in agreement with the values obtained by Zetasizer measurements).

### 3.2. Selectivity of GC-AuNPs for Fe^3+^ Ions and Interference Effects

**Figure 2:**
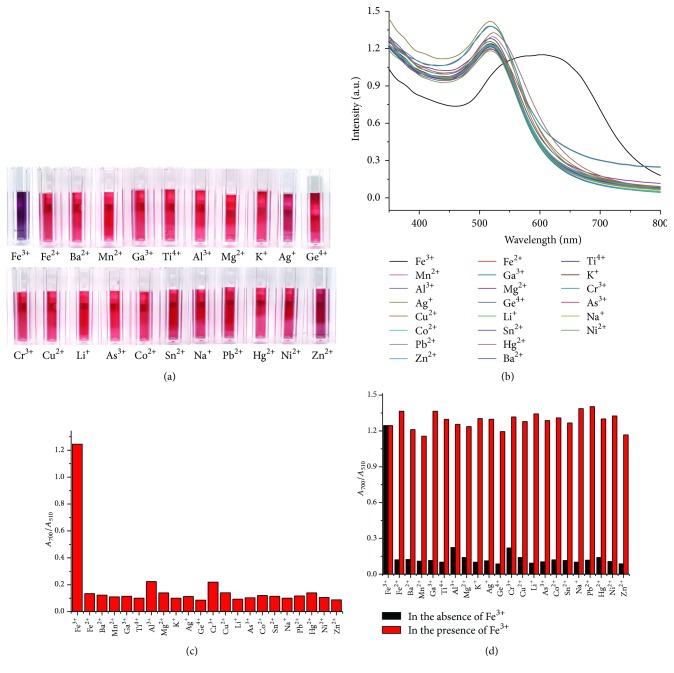
(a) Photographic color images, (b) UV-Vis absorption spectra, and (c) absorption ratios (*A*_700_/*A*_510_) of GC-AuNPs with 90 *μ*M Fe^3+^ and 900 *μ*M metal cations (Fe^2+^, Ba^2+^, Mn^2+^, Ga^3+^, Ti^4+^, Al^3+^, Mg^2+^, K^+^, Ag^+^, Ge^4+^, Cr^3+^, Cu^2+^, Li^+^, As^3+^, Co^2+^, Sn^2+^, Na^+^, Pb^2+^, Hg^2+^, Ni^2+^, and Zn^2+^ ions) at pH 6, 70°C, and 300 mM NaCl concentration. (d) Absorption ratios (*A*_700_/*A*_510_) of GC-AuNPs with 900 *μ*M metal cations (Fe^3+^, Fe^2+^, Ba^2+^, Mn^2+^, Ga^3+^, Ti^4+^, Al^3+^, Mg^2+^, K^+^, Ag^+^, Ge^4+^, Cr^3+^, Cu^2+^, Li^+^, As^3+^, Co^2+^, Sn^2+^, Na^+^, Pb^2+^, Hg^2+^, Ni^2+^, and Zn^2+^ ions) in the presence and absence of 90 *μ*M Fe^3+^.

The selectivity of the GC-AuNP sensor towards Fe^3+^ was investigated by using 90 *μ*M Fe^3+^ and 900 *μ*M other metal cations (Fe^2+^, Ba^2+^, Mn^2+^, Ga^3+^, Ti^4+^, Al^3+^, Mg^2+^, K^+^, Ag^+^, Ge^4+^, Cr^3+^, Cu^2+^, Li^+^, As^3+^, Co^2+^, Sn^2+^, Na^+^, Pb^2+^, Hg^2+^, Ni^2+^, and Zn^2+^ ions). It was found that, besides Fe^3+^, the other metal ions did not give any color change (Figure 2(a)). The absorption spectra of GC-AuNP solutions upon addition of various metal ions were recorded at pH 6, 70°C, and 300 mM NaCl concentration (Figure 2(b)), and the absorption band at 700 nm was uniquely strong for Fe^3+^, allowing its easy differentiation from other ions. The selectivity for Fe^3+^ ions was estimated by comparing of the absorbance ratio (*A*_700_/*A*_510_) of the solution containing Fe^3+^ ions with that of solutions containing other metal ions (Figure 2(c)). The reason why the absorbance ratio was calculated with the absorbance at 510 and 700 nm was that the absorption ratio (*A*_700_/*A*_510_) was linearly correlated to the Fe^3+^ concentration in the linear range of 0–180 *μ*M. A high absorbance ratio was attributed to aggregated AuNPs exhibiting a dark midnight blue color, whereas a lower ratio indicated well-dispersed GC-AuNPs. AuNPs were found to selectively react with Fe^3+^ ions, as indicated by the marked increase in the corresponding absorbance ratio. Specifically, the absorbance ratio of GC-AuNPs in the presence of Fe^3+^ was about 6–8 times higher than those obtained for other metal ions, suggesting a specific metal ion coordination between GC-AuNPs and Fe^3+^ ions. To investigate the interference effect of other metal ions on the selectivity of GC-AuNPs towards Fe^3+^, we examined the absorbance intensity of GC-AuNPs in the simultaneous presence of Fe^3+^ and other metal ions. The determination of Fe^3+^ ions was not affected by other metal ions, even when their concentration was ten times higher than that of Fe^3+^, because no metal ions except Fe^3+^ produced an absorption band at 700 nm (Figure 2(d)). Thus, other metal ions did not show any interference under the optimum Fe^3+^ sensing conditions, proving the excellent selectivity of the GC-AuNP probe for Fe^3+^ monitoring. The selectivity for Fe^3+^ ions was further tested using solutions containing various anions. Interestingly, among the tested anions, GC-AuNPs were found to react exclusively with iodide ions (Figure S1A in the Supporting Information available online at https://doi.org/10.1155/2017/3648564) among various anions. This interference was removed by the addition of a masking agent (SCN^−^) for iodide ions (Figure S1(B) in the Supporting Information) [36].

### 3.3. GC to AuNP and Fe^3+^ to GC-AuNP Binding Sites

**Figure 3:**
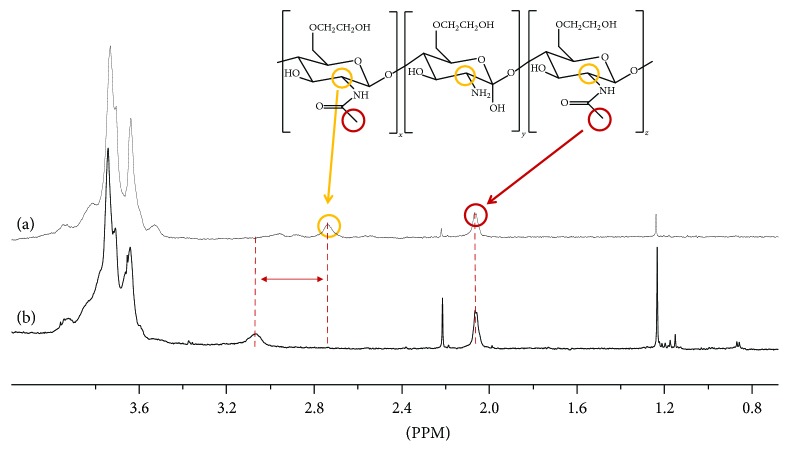
^1^H NMR spectra of (a) GC and (b) GC-AuNPs.

The binding sites of GC on the AuNPs were investigated by ^1^H NMR spectroscopy (Figure 3). The ^1^H NMR spectrum of free GC showed two characteristic peaks at 2.06 ppm (CH_3_ protons of the acetyl groups of* N*-acetamidoglucose units) and 2.60 ppm (CH proton of* N*-unsubstituted glucosamine units). The signal of GC at 2.60 ppm shifted to 3.06 ppm in the spectrum of GC-AuNPs, whereas that at 2.06 ppm did not shift (Figure 3(b)), indicating that the AuNPs were conjugated to the nitrogen atoms of GC, as expected [37].

**Figure 4:**
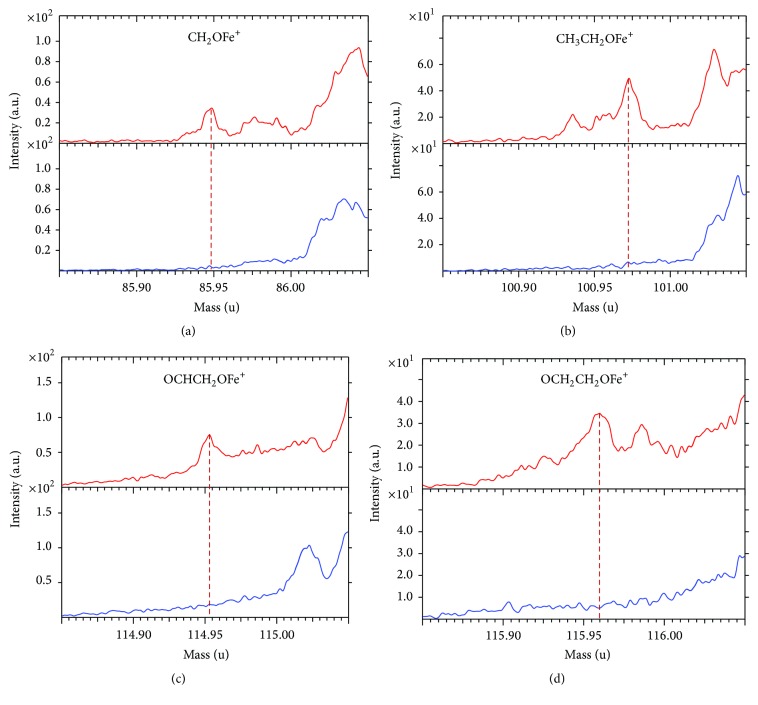
Mass peaks of (a) CH_2_OFe^+^, (b) CH_3_CH_2_OFe^+^, (c) OCHCH_2_OFe^+^, and (d) OCH_2_CH_2_OFe^+^ fragments in the TOF-SIMS spectrum of GC-AuNPs (blue) and Fe^3+^-GC-AuNPs (red). These molecular fragments were expected based on the Fe^3+^-GC-AuNP structural elements in the zoomed circle in Scheme 1.

To confirm the Fe^3+^-binding sites, TOF-SIMS spectra were recorded for GC-AuNPs and Fe^3+^-GC-AuNPs (Figure 4). The TOF-SIMS spectrum of Fe^3+^-GC-AuNPs displayed fragments corresponding to CH_2_OFe^+^, OCHCH_2_OFe^+^, CH_3_CH_2_OFe^+^, and OCH_2_CH_2_OFe^+^, whereas these were not observed in the corresponding spectrum of GC-AuNPs. These fragment patterns indicate that Fe^3+^ must be bound to the ethylene glycol oxygen atoms of GC [38, 39].

Next, we measured the XPS spectra of GC-AuNPs and Fe^3+^-GC-AuNPs to characterize the binding of Fe^3+^ ion to GC-AuNPs (Figure S2 in the Supporting Information). In the spectrum of Fe^3+^-GC-AuNPs, the high resolution Fe 2p_3/2_ signal at 710.6 eV is attributed to the binding energy of Fe-O bonds, indicating that the Fe^3+^ ions are bound to the oxygen atoms of GC [40].

### 3.4. Optimum Sensitivity Conditions for the GC-AuNP Sensor

**Figure 5:**
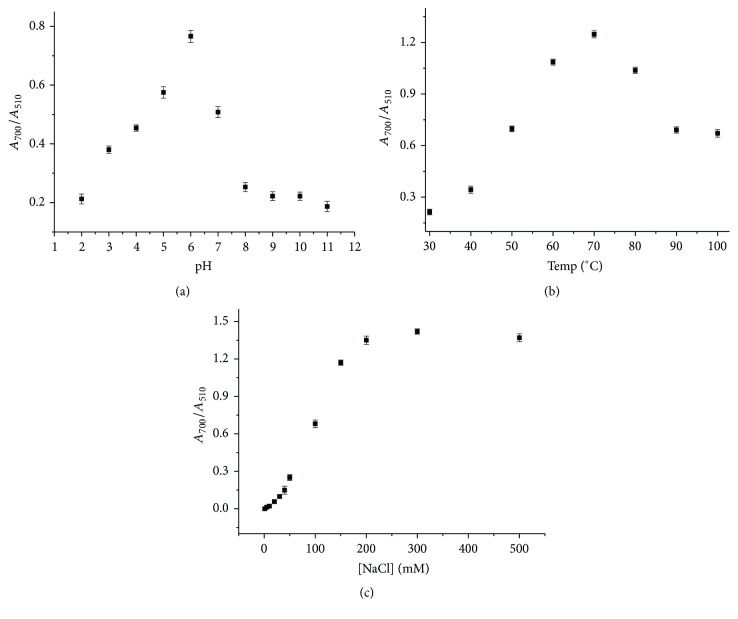
Absorption ratios (*A*_700_/*A*_510_) of Fe^3+^-GC-AuNPs as a function of (a) pH, (b) temperature, and (c) concentration of NaCl.

After establishing the good selectivity of GC-AuNPs towards Fe^3+^, the sensitivity of the GC-AuNP probe was examined as a function of pH, temperature, salt concentration, GC concentration, and reaction time. The performance of the GC-AuNP sensor towards Fe^3+^ was highly influenced by the pH of the medium. The absorbance ratio (*A*_700_/*A*_510_) changes of Fe^3+^-GC-AuNPs were examined in the range of pH 2–11, and it was found that this ratio dramatically increased at pH 6 (Figure 5(a)). Presumably, Fe^3+^ ions are optimally bound to the O atoms of GC in its O-hexadentate form at slightly acidic pH (pH 6), resulting in the highest sensitivity.

Next, the GC-AuNP aggregation in the presence of Fe^3+^ ions was tested in the temperature range of 30–100°C. Although the absorption ratio increased as the temperature was raised from 30 to 70°C, it significantly decreased from 70 to 100°C. GC conjugated to AuNP seemed to be perturbed structurally above 70°C. As a result, 70°C was chosen as the optimal temperature (Figure 5(b)).

The aggregation of GC-AuNPs in the presence of Fe^3+^ ions was optimized by increasing the NaCl concentration above ~50 mM. The absorption ratio increased until the salt concentration reached 300 mM (Figure 5(c)). As a result, this concentration was used, which resulted in the highest absorption ratio.

In order to determine the optimum GC concentration for conjugation to AuNPs a 0.4 *μ*g mL^−1^ Fe^3+^ solution was added to the sample, and the absorbance ratios were measured. The most suitable GC concentration for AuNP aggregation was found to be 30–35 *μ*M (data not shown).

Moreover, the aggregation kinetics of GC-AuNPs in the presence of various Fe^3+^ concentrations were evaluated by monitoring the absorbance ratios. The absorbance ratios continuously increased and reached a plateau at 40-min regardless of the Fe^3+^ concentration; thus, a 40-min reaction time was found to be necessary (Figure S3 in the Supporting Information).

### 3.5. Quantitation of Fe^3+^ Using the GC-AuNPs Sensor

**Figure 6:**
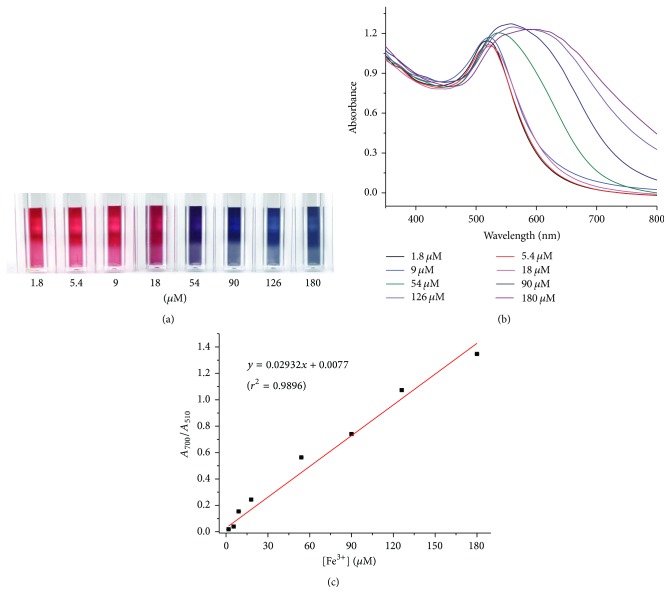
(a) Photographic images of the color change of GC-AuNPs upon Fe^3+^ addition at various concentrations (1.8, 5.3, 8.9, 17.9, 53.7, 89.5, 125.3, and 179.0 *μ*M from left to right) in the presence of 300 mM NaCl. (b) UV-Vis absorption spectra of GC-AuNPs upon addition of Fe^3+^ of various concentrations of Fe^3+^ (i.e., 1.8, 5.3, 8.9, 17.9, 53.7, 89.5, 125.3, and 179.0 *μ*M) in the presence of 300 mM NaCl. (c) Absorption ratios (*A*_700_/*A*_510_) of Fe^3+^-GC-AuNP versus Fe^3+^ concentration in the presence of 300 mM NaCl (*y* = 0.02932*x* + 0.0077 (*r*^2^ = 0.9896)).

The color change of GC-AuNPs correlates with the concentration of Fe^3+^ ions and can be monitored from the absorption ratio. The GC-AuNP solution color changed gradually from red to dark midnight blue as the Fe^3+^ concentration increased (Figure 6(a)). The absorbance increase at 700 nm and the concomitant decrease of the peak at 510 nm were measured for increasing Fe^3+^ concentrations (1.8, 5.3, 8.9, 17.9, 53.7, 89.5, 125.3, and 179.0 *μ*M) in GC-AuNP solutions (Figure 6(b)). The absorbance ratio at each Fe^3+^ ion concentration was determined in triplicate (Figure 6(c)) within the linear dynamic range of the calibration curve (0.0–180 *μ*M). Linear regression analysis of the calibration curve displayed good linearity with a regression coefficient of 0.9834. The limits of detection (LODs) of this colorimetric probe were calculated to be 11.3, 29.2, and 46.0 nM for tap water, pond water, and iron supplements, respectively, using (3*σ*/slope).

### 3.6. Application of the GC-AuNP Probe in the Analysis of Real Samples

This colorimetric method was validated by determination of Fe^3+^ in real tap water, pond water, and iron supplement samples. Tap water, pond water, and iron supplement samples added with 0.6 and 1.8 *μ*M Fe^3+^ were analyzed using the GC-AuNP probe and by AAS. The results of the GC-AuNP probe assay are in good agreement with those obtained by AAS, as shown in Table 1.

As can be seen from Table 2, no Fe^3+^ ions were detected in real water samples by both the colorimetric AuNP probe and AAS. Therefore, the present assay method for the detection of Fe^3+^ ions in aqueous samples would be superior to the currently used AAS method in terms of facileness, sensitivity, and cost.

It should be noted that the present probe provides the lowest LOD for the analysis of Fe^3+^ ions in aqueous samples as compared to the previously reported nanoparticle sensors for the detection of Fe^3+^ ions, as shown in Table 3.

## 4. Conclusions

AuNPs conjugated with GC were prepared and used for the selective and highly sensitive colorimetric detection of Fe^3+^. The sensing mechanism of this colorimetric probe is based on the aggregation of GC-AuNPs in the presence of Fe^3+^ ions, which were found to be coordinated to the ethylene glycol oxygen atoms of GC conjugated to the nanoparticles.

This method offers a simple, highly sensitive, highly selective, and inexpensive on-site monitoring of Fe^3+^ ions, allowing detection of concentrations as low as 11.3 nM to be achieved within 40 min.

## Supplementary Material

Fig. S1: (A) Absorption ratios (A_700_/A_510_) of GC-AuNPs with 90 μM Fe^3+^ and 900 μM anions. (B) Absorption ratios (A_700_/A_510_) of GC-AuNPs with 90 μM Fe^3+^ and 900 μM anions in the presence of the masking agent SCN^−^. Fig. S2: (A) XPS spectra of GC-AuNPs and Fe^3+^-GC-AuNPs. (B) Fe 2p_3/2_ signal in Fe^3+^-GC-AuNPs at 710.6 eV. Fig. S3: Absorbance ratio (A_700_/A_510_) as a function of time over 60 min.

## Figures and Tables

**Table 1:** Determination of Fe^3+^ ions in spiked tap water, pond water, and iron supplement samples analyzed by the GC-AuNP probe and AAS.

Amount of Fe^3+^ added to real samples (*n* = 7)						
Sample	Present probe					AAS
	Added amount (nM)	Detected amount (nM)	Coefficient of variation (%)	Recovery (%)	LOD (nM)	Detected amount (nM)
Tap water	60.0	60.4 ± 0.745	1.23	100.7 ± 1.24	11.3	60.0 ± 1.00 × 10^−2^
	1.80 × 10^2^	1.81 × 10^2^ ± 0.472	0.261	100.6 ± 0.262		1.80 × 10^2^ ± 1.00 × 10^−2^
Pond water	60.0	60.3 ± 0.429	0.711	100.6 ± 0.715	29.2	60.0 ± 1.00 × 10^−2^
	1.80 × 10^2^	1.80 × 10^2^ ± 0.413	0.229	100.1 ± 0.229		1.80 × 10^2^ ± 1.00 × 10^−2^
Iron supplement	60.0	60.4 ± 0.629	1.04	100.7 ± 1.05	46.0	60.0 ± 1.00 × 10^−2^
	1.80 × 10^2^	1.79 × 10^2^ ± 1.05	0.589	99.6 ± 0.587		1.80 × 10^2^ ± 1.00 × 10^−2^

**Table 2:** Determination of Fe^3+^ in real water samples collected from KIST.

Sample	Content of Fe^3+^ ion (*μ*M)	
	Present probe	AAS
Tap water	<0.01	<0.1
Pond water	<0.01	<0.1

**Table 3:** Comparison of present probe with previously reported nanoparticle assay methods for the analysis of Fe^3+^.

Detection tool	Functionalized agent	Sensing method	LDR^a^	LOD^b^	Ref
Chemosensor	2-Chloro-N-(9-ethyl-9H-carbazol-3-yl)acetamide and 2-(((pyridin-2-ylmethyl)amino)methyl) phenol	Color change	—	13.5 *μ*M	[14]
Chemosensor	2-(Aminomethyl)benzimidazole dihydrochloride and 4-diethylaminosalicylaldehyde	Color change	0–5.0 *μ*M	1.21 *μ*M	[15]
Chemosensor	5-Amino-1H-imidazole-4-carboxamide and 8-hydroxyjulolidine-9-carboxaldehyde	Fluorescence	—	0.27 *μ*M	[16]
Cu nanoclusters	Polyethylenimine	Fluorescence	0.5–1000 *μ*M,	0.34 *μ*M	[19]
Au nanoclusters	L-3,4-Dihydroxyphenylalanine	Fluorescence	5–1280 *μ*M	3.5 *μ*M	[20]
AgNPs	N-Acetyl-L-cysteine	Color change (etching)	80 nM–80 mM	80 nM	[21]
Au nanorods	Label-free	Color change (etching)	—	1.79 *μ*M	[22]
Graphene quantum dots	1-Butyl-3-methylimidazolium hexafluorophosphate (BMIMPF_6_)	Fluorescence	0–80 *µ*M	7.22 *µ*M	[23]
Carbon dots	label-free	Fluorescence	0–20 *μ*M.	0.32 *μ*M	[24]
AgNPs	Calix[4] resorcinarene polyhydrazide	Fluorescence	0.1–10 *μ*M	0.1 *μ*M.	[25]
AuNPs	Pyrophosphate	Color change (aggregation)	10–60 *μ*M	5.6 *μ*M.	[26]
AuNPs	Histidine	Color change (aggregation)	—	—	[27]
AuNPs	p-Amino salicylic acid dithiocarbamate	Color change (aggregation)	40–80 mM	14.82 nM	[28]
AuNPs	Glycol chitosan	Color change (aggregation)	0.0–180 *μ*M	11.3 nM	This work

^a^LDR; linear dynamic range; ^b^LOD; limit of detection.
