# Supplementary material for: Highly Sensitive Colorimetric Assay for Determining Fe3+ Based on Gold Nanoparticles Conjugated with Glycol Chitosan
Source: J Anal Methods Chem. 2017 May 23;2017:3648564. doi: 10.1155/2017/3648564 (PMC5463166; doi:10.1155/2017/3648564)
Supplement: Supplementary file 1 — Fig. S1: (A) Absorption ratios (A700/A510) of GC-AuNPs with 90 μM Fe3+ and 900 μM anions. (B) Absorption ratios (A700/A510) of GC-AuNPs with 90 μM Fe3+ and 900 μM anions in the presence of the masking agent SCN−. Fig. S2: (A) XPS spectra of GC-AuNPs and Fe3+-GC-AuNPs. (B) Fe 2p3/2 signal in Fe3+-GC-AuNPs at 710.6 eV. Fig. S3: Absorbance ratio (A700/A510) as a function of time over 60 min. [file 3648564.f1.docx]

**Supplementary Materials**

**Fig. S1**

**(A)**


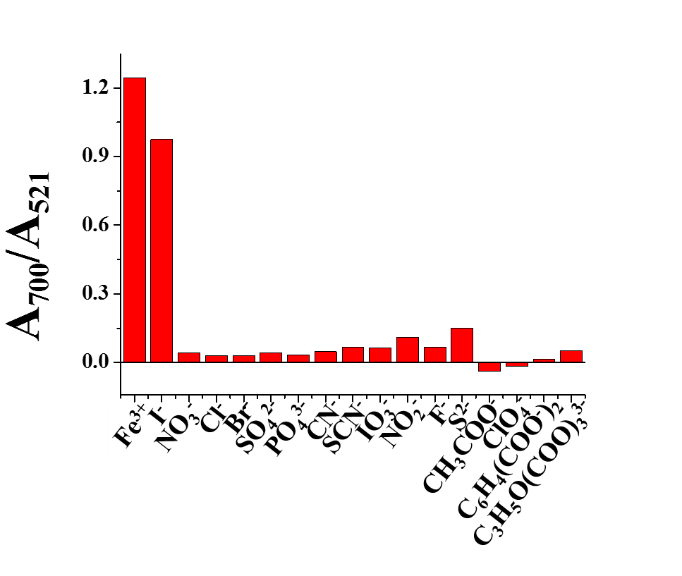


**(B)**


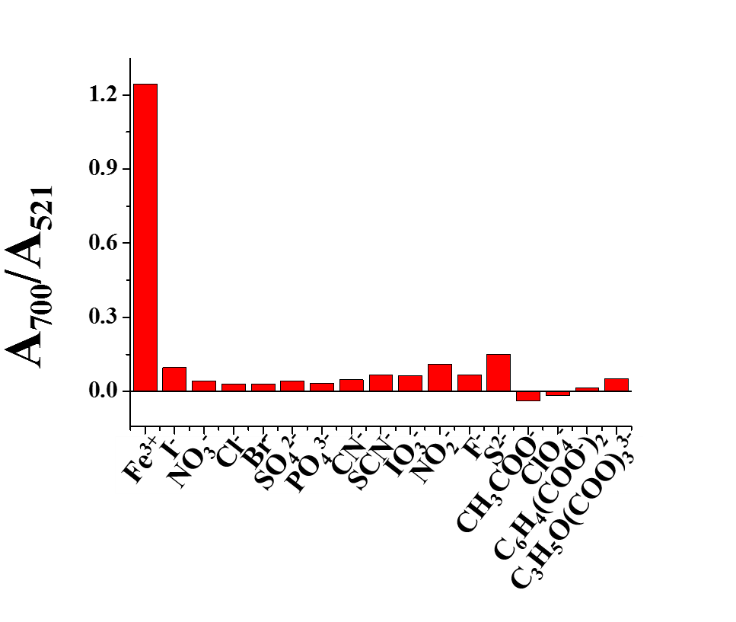


**Fig. S1.** (A) Absorption ratios (A_700_/A_510_) of GC-AuNPs with 90 μM Fe^3+^ and 900 μM anions (I^−^, NO_3_^−^, Cl^−^, Br^−^, SO_4_^2−^, PO_4_^3−^, CN^−^, SCN^−^, IO_3_^−^, NO_2_^−^, F^−^, S^2−^, CH_3_COO^−^, ClO_4_^−^, C_6_H_4_(COO^−^)_2_, and C_3_H_5_O(COO)_3_^3−^ ions) at pH 6, 70 °C, and 300 mM NaCl concentration. (B) Absorption ratios (A_700_/A_510_) of GC-AuNPs with 90 μM Fe^3+^ and 900 μM anions under the same conditions in the presence of the masking agent SCN^−^.

**Fig. S2**

**(A)**

**
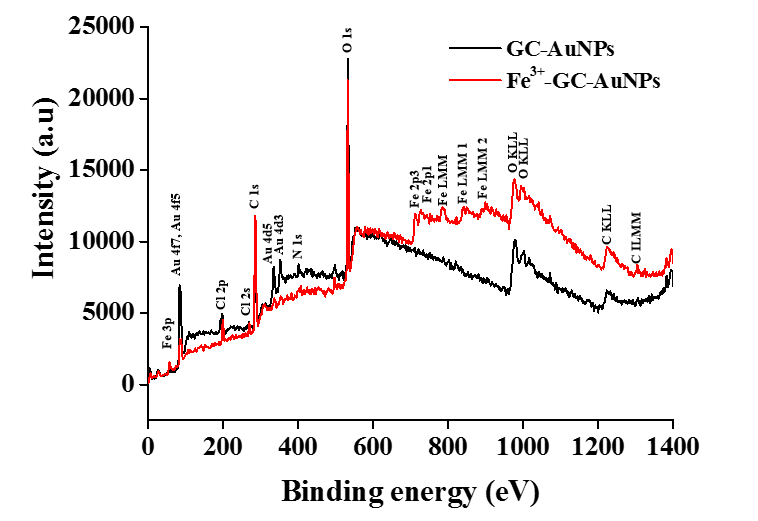
**

**(B)**

**
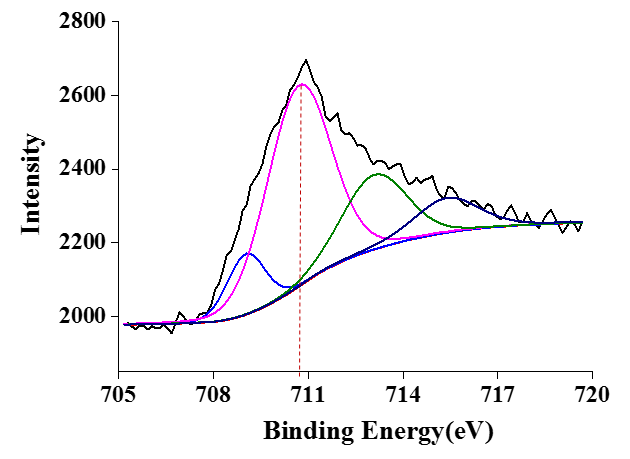
**

**Fig. S2.** (A) Wide scan XPS spectra of GC-AuNPs (black line) and Fe^3+^-GC-AuNPs (red line); (B) Fe 2p_3/2_ signal in Fe^3+^-GC-AuNPs at 710.6 eV, attributed to the Fe-O binding energy in Fe^3+^-GC-AuNPs.

**Fig. S3**

**
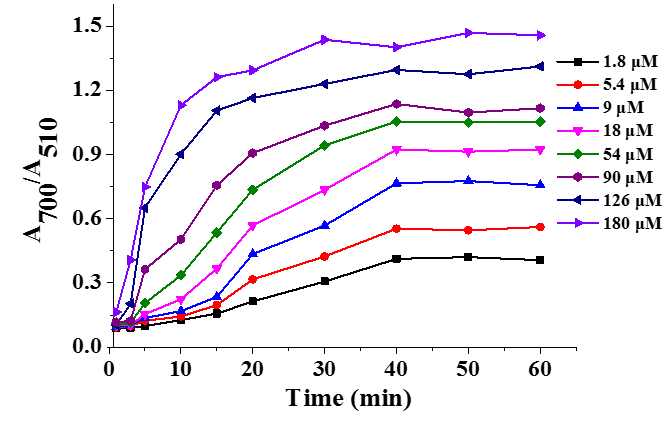
**

**Fig. S3.** Absorbance ratio (A_700_/A_510_) as a function of time over 60 min in the presence of various concentrations of Fe^3+^.
